# Evidence of region‐wide bat population decline from long‐term monitoring and Bayesian occupancy models with empirically informed priors

**DOI:** 10.1002/ece3.5612

**Published:** 2019-09-11

**Authors:** Thomas J. Rodhouse, Rogelio M. Rodriguez, Katharine M. Banner, Patricia C. Ormsbee, Jenny Barnett, Kathryn M. Irvine

**Affiliations:** ^1^ National Park Service and Human and Ecosystem Resiliency and Sustainability Lab Oregon State University‐Cascades Bend OR USA; ^2^ Human and Ecosystem Resiliency and Sustainability Lab and Northwestern Bat Hub Oregon State University‐Cascades Bend OR USA; ^3^ Department of Mathematical Sciences Montana State University Bozeman MT USA; ^4^ Willamette National Forest Springfield OR USA; ^5^ Mid‐Columbia River National Wildlife Refuge Complex U.S. Fish and Wildlife Service Burbank WA USA; ^6^ Northern Rocky Mountain Science Center US Geological Survey Bozeman MT USA; ^7^ Present address: Human and Ecosystem Resiliency and Sustainability Lab Oregon State University‐Cascades 1500 SW Chandler Ave. Bend OR 97702 USA

**Keywords:** acoustic recording units, Chiroptera, extinction risk, monitoring, North American Bat Monitoring Program, population decline, trend, ultrasonic acoustic detectors

## Abstract

Strategic conservation efforts for cryptic species, especially bats, are hindered by limited understanding of distribution and population trends. Integrating long‐term encounter surveys with multi‐season occupancy models provides a solution whereby inferences about changing occupancy probabilities and latent changes in abundance can be supported. When harnessed to a Bayesian inferential paradigm, this modeling framework offers flexibility for conservation programs that need to update prior model‐based understanding about at‐risk species with new data. This scenario is exemplified by a bat monitoring program in the Pacific Northwestern United States in which results from 8 years of surveys from 2003 to 2010 require updating with new data from 2016 to 2018. The new data were collected after the arrival of bat white‐nose syndrome and expansion of wind power generation, stressors expected to cause population declines in at least two vulnerable species, little brown bat (*Myotis lucifugus*) and the hoary bat (*Lasiurus cinereus*). We used multi‐season occupancy models with empirically informed prior distributions drawn from previous occupancy results (2003–2010) to assess evidence of contemporary decline in these two species. Empirically informed priors provided the bridge across the two monitoring periods and increased precision of parameter posterior distributions, but did not alter inferences relative to use of vague priors. We found evidence of region‐wide summertime decline for the hoary bat ($\hat{\lambda }$ = 0.86 ± 0.10) since 2010, but no evidence of decline for the little brown bat ($\hat{\lambda }$ = 1.1 ± 0.10). White‐nose syndrome was documented in the region in 2016 and may not yet have caused regional impact to the little brown bat. However, our discovery of hoary bat decline is consistent with the hypothesis that the longer duration and greater geographic extent of the wind energy stressor (collision and barotrauma) have impacted the species. These hypotheses can be evaluated and updated over time within our framework of pre–post impact monitoring and modeling. Our approach provides the foundation for a strategic evidence‐based conservation system and contributes to a growing preponderance of evidence from multiple lines of inquiry that bat species are declining.

## Graphical abstract

We evaluate evidence of region‐wide summertime bat population decline in the Pacific Northwestern United States over a 16‐year period with large‐sample acoustic monitoring survey data and Bayesian occupancy models with empirically informative parameter priors. We provide compelling evidence that the hoary bat (shown in the photo, credit Michael Durham), a species known to be at risk from collision and barotrauma at wind energy production facilities, is experiencing regional decline at a rate of approximately 2% per year since 2010. Our study outlines a hypothesis‐driven model‐based framework for linking monitoring to conservation decision‐making.

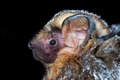

## 1 INTRODUCTION

Evidence‐based conservation of at‐risk species is challenged by lack of information about population trends over time, particularly for those species that are cryptic and difficult to survey. In situations where directly counting individual organisms is infeasible, occupancy modeling of detection/nondetection survey data provides an alternative to abundance models for detecting regional‐scale population declines (Jones, 2011; MacKenzie et al., 2002; Noon, Bailey, Sisk, & McKelvey, 2012). Multi‐season occupancy models (e.g., MacKenzie, Nichols, Hines, Knutson, & Franklin, 2003; Royle & Kery, 2007) support inferences about changing occupancy probabilities and dynamic site turnover parameters over time. These parameters reflect changes in species distribution but are also expected to reflect the underlying latent changes in population size (Gaston et al., 2000; Holt, Gaston, & He, 2002; Zuckerberg, Porter, & Corwin, 2009) and extinction risk (Noon et al., 2012), albeit with some amount of elasticity (e.g., Kery & Royle, 2016; Royle & Kery, 2007; Steenweg, Hebblewhite, Whittington, Lukacs, & McKelvey, 2018). When harnessed to a Bayesian inferential paradigm, this modeling framework offers considerable flexibility for regional conservation monitoring programs that need to update prior model‐based understanding with new data as they become available (e.g., Dorazio & Johnson, 2003; Ellison, 2004). Rather than starting anew after each cycle of data collection, model‐fitting, evaluation, and inference, Bayes theorem allows for previous modeling results, in the form of posterior probability distributions, to be used as prior probability distributions that formally represent best‐available understanding about model parameters (Crome, Thomas, & Moore, 1996; Hobbs & Hooten, 2015; McCarthy & Masters, 2005). With new data, this prior understanding can in turn be updated and represented as new, updated posteriors, with an expectation that clarity about population distribution and abundance, in the form of precision, will increase over time (Morris, Vesk, McCarthy, Bunyavejchewin, & Baker, 2015). In this way, the empirically informative Bayesian inferential paradigm, when harnessed to replicate geographically extensive large‐sample encounter surveys, provides a way to “scaffold”, or build upon, prior knowledge to improve conservation decision‐making.

**Figure 1:**
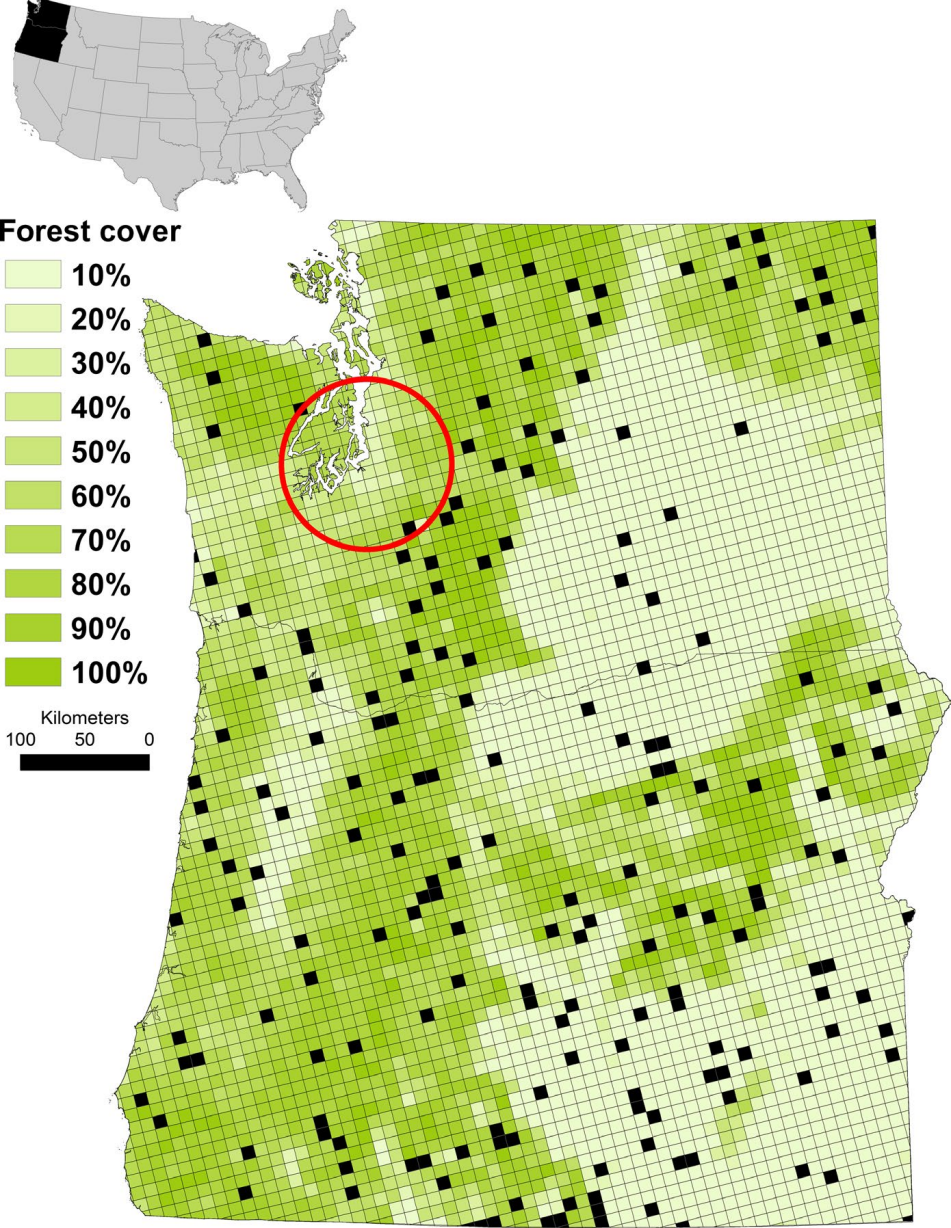
The study area, Oregon and Washington, USA, overlaid with the grid‐based sampling frame, average % forest cover of each frame sample unit (grid cell), and the 190 sample units surveyed during 2016–2018 (black squares) that follow a spatially balanced master sample design. The area where white‐nose syndrome has been confirmed circa 2019 is circled in red

This scenario is exemplified by a bat monitoring program in an ~440,000 km^2^ region of the Pacific Northwestern United States (Figure 1) in which the occupancy modeling results from 8 years of monitoring, which ended in 2010 (Rodhouse et al., 2012, 2015), require updating with new survey data gathered during 2016–2018 for contribution to the North American Bat Monitoring Program (NABat; Loeb et al., 2015). There is urgency to this opportunity to scaffold upon prior information because bat populations in the region are facing potentially catastrophic declines (e.g., O'Shea, Cryan, Hayman, Plowright, & Streicker, 2016) from the recent arrival of the bat disease white‐nose syndrome (Lorch et al., 2016) and the rapidly expanding footprint of the wind power industry (Arnett et al., 2016). The cumulative impacts by these novel threats are likely exacerbated by accelerated environmental changes (Jones, Jacobs, Kunz, Willig, & Racey, 2009; Jung & Threlfall, 2016), including global entomofauna die‐off (Sanchez‐Bayo & Wyckhus, 2019), which is particularly worrisome given that the majority of North American bat species are insectivorous. In general, there is a global paucity of empirical knowledge about bat population trends and fewer still that evaluate trends over broad regions and long time periods (Jones et al., 2009). But there is growing evidence that many species are experiencing evolutionarily unprecedented, massive declines (O'Shea et al., 2016). Our emphasis on geographically extensive regional inference is noteworthy because bats are so vagile that a local‐scale decline, for example one detected within a small national park, is difficult to interpret and use to motivate conservation without broader context (e.g., via replication elsewhere).

Here, we ask whether there is evidence of regional summertime decline in the northwestern United States after three additional years of surveys for two vulnerable species, the little brown bat (*Myotis lucifugus*) and the hoary bat (*Lasiurus cinereus*). We focus on the little brown bat because it has been listed as threatened in Canada (Committee on the Status of Endangered Wildlife in Canada (COSEWIC), 2013) and considered for similar protection in the United States (Federal Register, 2015) following precipitous declines in eastern North America from white‐nose syndrome (Dzal, McGuire, Veselka, & Fenton, 2011; Frick et al., 2010) and because the disease was first confirmed in the northwestern portion of our study region (Figure 1) in 2016 from a dead little brown bat (Lorch et al., 2016). We focus on the hoary bat because it is the most frequently encountered species in carcass recoveries at wind power generation facilities in many regions of North America and thought to be at risk of widespread decline (Arnett et al., 2016; Cryan & Barclay, 2009; Frick et al., 2017). We build upon the same dynamic occupancy model used by Rodhouse et al., (2015) and use their 2010 posterior estimates to create empirically informed priors as a way to formally incorporate best‐available information about occupancy parameters into an updated assessment of decline.

## 2 METHODS

### 2.1 Study area and biogeographic gradients

We monitored bats during summer (June–September) via coordinated acoustic surveys across Oregon and Washington states, in the northwestern region of the United States (Figure 1). The region is divided in half by the north–south trending Cascade Range that creates a distinct rain shadow over the eastern half of the region and a west‐to‐east forest cover gradient that is a dominant biogeographic influence on bats (Figure 1). The forest cover gradient in the region is strongly correlated with net primary productivity (*ρ* = 0.7) and moderately so with precipitation and elevation (Rodhouse et al., 2012, 2015). The little brown bat and hoary bat range widely across the region and are found in all habitat types but are associated with forested landscapes more than nonforested shrub steppe (Hayes, 2003; Kalcounis‐Rüppell, Psyllakis, & Brigham, 2005; Rodhouse et al., 2015). Forests and also topographic roughness (*SD* of elevation) provide the keystone structures (sensu Tews et al., 2004; e.g., live and dead standing trees, crevices in large cliffs) used by bats for summer and winter roosting that are additional biogeographic drivers of bat distributional patterns in the region (Humphrey, 1975; Pierson, 1998; Rodhouse et al., 2015). Forest cover (% of sample unit classified as any forest type), elevation (sample unit mean), 30‐year mean annual precipitation (sample unit mean), and topographic roughness (*SD* of sample unit elevation) were included as occupancy model covariates both during initial modeling by Rodhouse et al., (2015) and in the present study.

### 2.2 Study survey design

Our study protocol is described in detail by Rodriguez et al. (2019). We used a grid‐based sampling frame of 100‐km^2^ square cells mapped across the study area to structure surveys and analyses (Figure 1). In 2003–2010 (Period 1), a combination of capture and acoustic surveys was conducted across the region in 241 grid cells (see Rodhouse et al., 2015, p. 1404). In 2016–2018 (Period 2), acoustic surveys were conducted in 190 grid cells, informed by a statistical power analysis (Banner, Irvine, Rodhouse, Donner, & Litt, 2019; Figure 1). During Period 1, grid cells were selected using a combination of constrained simple random sampling and nonrandom contributions from land management agencies and researchers using compatible methodology (see Rodhouse et al., 2015 for additional details). During Period 2, grid cells were selected using the NABat spatially balanced (via the Generalized Random Tessellation Stratified design; Rodhouse et al., 2012; Rodhouse, Vierling, & Irvine, 2011; Stevens & Olsen, 2004) randomized master sample (Larsen, Olsen, & Stevens, 2008; Loeb et al., 2015). Approximately 80% (*n* = 155) of the 190 grid cells surveyed during Period 2 were chosen following the spatially balanced order of the master sample. Twenty per cent were chosen from the Period 1 legacy sample in order to provide spatio‐temporal overlap between the two periods. This was less than the rule‐of‐thumb threshold suggested by Irvine, Rodhouse, Wright, and Olsen (2018) that, if exceeded, would require a more complex likelihood weighting in subsequent modeling in order to mitigate for an unrepresentative sample. This large (*n* = 190) and spatially balanced random sample is representative of the region of interest and supports robust scope of inference.

Spatially replicated within‐season (June–September) single‐night surveys were conducted in grid cells. Multiple‐night replicates were avoided in order to maintain backward compatibility with the Period 1 revisit design and because Wright, Irvine, and Rodhouse (2016; and others, see Hayes, 1997) found evidence of serial correlation suggesting a lack of independence in bat activity among consecutive nights. Numbers of within‐season revisits ranged from 1 to 12 per season in Period 1 and were standardized to four visits during Period 2. Surveys during Period 1 consisted of mist net capturing and/or recording of bats with Pettersson D240x and D500x ultrasonic detectors (Pettersson Elektronik) along watercourses. Survey method was included as a detection model covariate during initial modeling by Rodhouse et al. (2015). Period 2 surveys were conducted only by recording bats with Pettersson D500x ultrasonic detectors. Duration of surveys varied during Period 1 from 2 hr to overnight, but lasted all night during Period 2. Duration was included as a detection model covariate for the Period 1 model. Survey date was included as a detection model covariate for both periods. Species identification methods from captures and bat call recordings used during Period 1 were described in detail by Rodhouse et al., (2015), but included the use of version 3 of the Sonobat software program (Sonobat; https://sonobat.com/) to process and assign call files to species and ad hoc manual verification by a single expert (J. Szewczak). During survey Period 2, all call files were processed and assigned to species using version 4 of Sonobat and also verified manually by a single expert (R. Rodriguez) but that followed the REMOVE workflow strategy outlined by Banner et al. (2018, p. 6147) to remove all false‐positive identification error from the data set prior to analysis so that the standard (false‐negative only) occupancy model could be used. Manual verification was conducted specifically to eliminate false‐positive errors by carefully examining highest‐quality call files used to make species detection decisions from each survey (e.g., focusing only on the few decision‐pivotal call files per species per survey night). Only the unambiguous call files assigned to little brown bat and hoary bats were used as evidence for detection. This REMOVE verification strategy is inherently conservative and elevates false‐negative error but our false‐negative errors (detection probabilities) were still acceptable (>40%, see Section 3) to obtain unbiased occurrence model parameter estimates.

### 2.3 Statistical analysis

We analyzed survey data from Period 2 only, using the results (specifically the estimated posterior mean and precision from occupancy model parameters) from Period 1 to construct empirically informative priors. Detection history matrices containing 190 rows and 12 columns (four single‐night visits per season) were constructed for Period 2, with matrix elements assigned a 1 for unambiguous detection or 0 otherwise. We used the same autoregressive multi‐season occupancy model (Royle & Dorazio, 2008) for Period 2 as for Period 1 presented by Rodhouse et al., (2012, 2015). Drawing on the Royle and Dorazio, (2008) autoregressive parameterization of the dynamic occupancy model, the initial occupancy state *z*(*i*,*t*) for sample unit (grid cell) *i* in the first year (*t* = 1) of sampling was modeled as.

*z*(*i*,1) ~ Bernoulli(Ψ_1_ *_i_*) for *i* = 1,…, *n*, with logit(Ψ_1_ *_i_*) = *β*_0_ + *β*_1_ForestCover*_i_* + *β*_2_Elevation*_i_* + *β*_3_Precipitation*_i_* + *β*_4_Topographic Roughness*_i_*. Subsequent survey years (*z*[*i*,*t*] for *t* = 2 and 3) were modeled conditional on the previous state, *z*(*i*,*t*)|*z*(*i*,*t*−1) ~ Bernoulli{π*_ti_*}, with logit(π*_ti_*) = *a_t_* + *b_t_* *z*(*i*,*t*−1) + *β*_1_ForestCover*_i_* + *β*_2_Elevation*_i_* + *β*_3_Precipitation*_i_* + *β*_4_Topographic Roughness*_i_*. The four environmental covariates were mean‐centered and standardized for computational efficiency and so that interpretation of derived parameters could be made at average environmental conditions (i.e., when coefficients were 0). The derived parameters *ϕ_t_* = logit^−1^(*a_t_* + *b_t_*) represented the probability of a unit remaining occupied by a species (e.g., survival) and *γ_t_* = logit^−1^(*a_t_*) the probability of a unit becoming newly occupied (e.g., colonization) for each given time step (*t*−1 to *t*). The occupancy probabilities in years *t* = 2,…,*T* were calculated recursively as Ψ*_t_* = Ψ*_t_*_−1_ *ϕ* *_t_* + (1 − Ψ*_t_*_−1_)*γ* *_t_*. We used the total unit occurrence growth rate over Period 2, *λ* = Ψ_2018_/Ψ_2016_, as our trend metric. Given mean‐centering of covariates, *λ* is interpreted as an overall region‐wide measure of net decline. Exploration of how derived parameter values vary along the environmental gradients could be accomplished by plugging in different covariate values (i.e., at high and low elevations), which we do by obtaining posterior distributions of Ψ_2018,_ *_i_* for each of the 4,500 grid cells in the study region and mapping posterior means to show an updated species distribution map of region‐wide occurrence probabilities for comparison with the 2010 map. We used a simpler detection model than Rodhouse et al. (2015), including survey date as a covariate but no additional covariates for method and duration, given the survey design standardization of those two variables during Period 2, where *y_j_*(*i*,*t*) | *z*(*i*,*t*) ~ Bernoulli {*p_i_*_,_ _t_* *z*(*i*,*t*)}and logit(*p_i_*_,_ *_t_*) = *α*_0_ + *α*_1_date*_i_*_,_ *_t_*_,_.

Given the differences in the survey methodology and call processing and species identification workflow, we only used vague Normal(0,10) priors for detection‐level parameters, effectively fitting our detection model without prior knowledge (i.e., from “scratch”). We used independent, empirically informed priors on the occupancy‐level parameters [**β**, *a_t_*, *b_t_*]. Informative priors were specified as Normal distributions with mean and standard deviation based on the posterior distributions estimated from the final year (2010) of Period 1 models provided by Rodhouse et al., (2015; Table 1). We compared our results with the same model but where vague priors (Normal[0,10]) were used instead. Vague priors, also referred to as uninformative or weakly informative priors (Northrup & Gerber, 2018), are regularizing priors (Gelman, Simpson, & Betancourt, 2017) that stabilize the posterior distributions for parameters {**β**, *a_t_*_−1_, *b_t_*_−1_} within a reasonable range on the logit scale but do not represent any substantive knowledge about their values a priori.

**Table 1:** Posterior distribution means and standard deviations from Period 1 (2010) used as empirically informed priors for Period 2 (2016–2018) models

Parameters	Little brown bat	Hoary bat
*β*_0_	3.53 ± 1.62	0.15 ± 1.15
*α*	0.14 ± 1.57	−0.68 ± 1.52
*β*	3.49 ± 1.76	4.32 ± 1.94
*β*_elevation_	−0.29 ± 0.27	−0.52 ± 0.29
*β*_precipitation_	1.59 ± 0.97	−0.41 ± 0.30
*β*_topographic roughness_	0.00 ± 0.29	−0.08 ± 0.21
*β*_forest_	0.46 ± 0.34	0.64 ± 0.26

**Figure 2:**
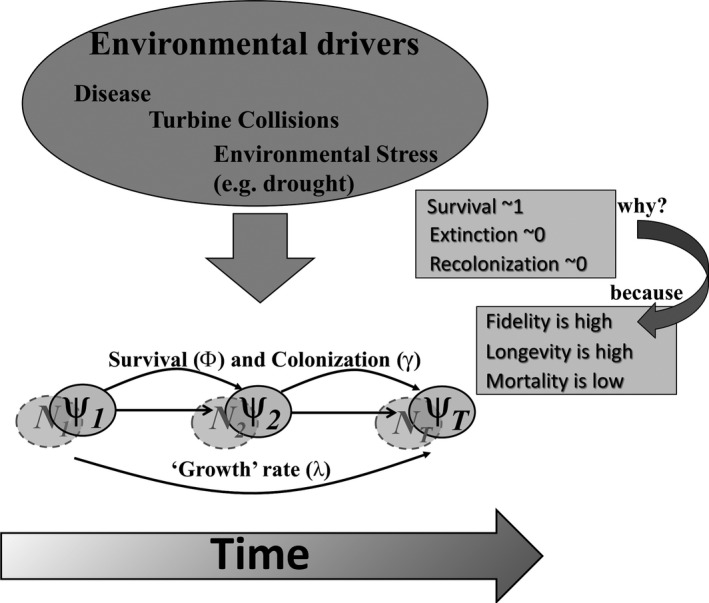
Conceptual diagram of occurrence state change (superimposed over latent abundance *N*) over time as a function of survival, recolonization, and extinction of sample unit occurrences from 1 year to the next. The net result of change can be characterized by the occurrence growth rate λ. The diagram outlines (right) hypothesized expectations for background rates of these parameters, drawing on knowledge of temperate‐zone bat life history strategies, but suggests extrinsic environmental drivers (e.g., disease, top of diagram) may alter these background rates, elevating adult bat mortality rates

In Figure 2, we conceptualize this model parameterization as hypothesized inter‐annual change in occurrence states (and in latent abundance), as a conditional Markov process governed by the dynamic rate parameters of sample unit occurrence survival (*ϕ*) and recolonization (*γ*), summarized by *λ*. We expect the background rates for these dynamic parameters to be stable and near 1 for *ϕ* and near 0 for *γ* because of the slow life history strategies of bats (low fecundity, adult longevity, and low adult mortality; Barclay & Harder, 2003; O'Shea et al., 2016; Promislow & Harvey, 1990) and high site fidelity (e.g., Barclay & Brigham, 2001; Lewis, 1995). We expect that novel extrinsic factors, particularly white‐nose syndrome (for little brown bat) and widespread wind energy development and associated collision and barotrauma (for hoary bat) will influence those dynamic rate parameters (O'Shea et al., 2016), reflected in declining $\hat{\psi }$ and $\hat{\lambda }$ < 1.

We used OpenBUGS 3.2.3 (Lunn, Spiegelhalter, Thomas, & Best, 2009), launched from R 3.5.1 (R Core Team, 2018) with the R2OpenBUGS library (Sturtz, Ligges, & Gelman, 2005) to implement Bayesian estimation of model parameters via Markov chain Monte Carlo (MCMC) samples from posterior distributions. Posterior summaries were based on 10,000 MCMC samples of the posterior distributions from three chains run simultaneously, thinned by a factor of 3, following an initial burn‐in of 5,000 MCMC iterations. We assessed convergence of MCMC chains with trace plots and the Gelman‐Rubin diagnostic,; convergence was reached for all parameters according to the criteria $|\hat{R}-1|<0.1$. We evaluated prior sensitivity by comparing inference and by examining vague and informative prior and posterior density plots. We evaluated model predictive performance with posterior summaries of the area under the curve of the receiver operating characteristic (AUC; Zipkin, Campbell Grant, & Fagan, 2012) and compare against summaries provided by Rodhouse et al., (2015). We evaluated evidence of residual spatial autocorrelation by estimating the Moran's *I* statistic for the occupancy residuals (Wright, Irvine, & Higgs, 2019) at distance thresholds from 10 km (adjacent neighbors) to 50 km. Our spatially balanced master sample design reduced spatial proximity of sample units, and we found no evidence of autocorrelation.

## 3 RESULTS

**Figure 3:**
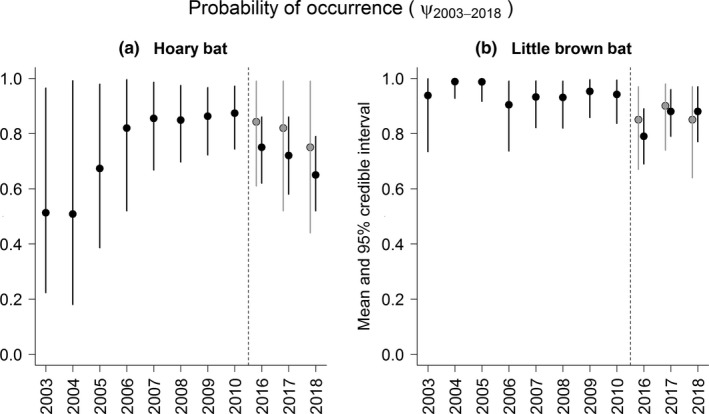
Posterior mean and 95% credible intervals for $\hat{\psi }$ from models fit to (a) hoary bat (*Lasiurus cinereus*) and (b) little brown bat (*Myotis lucifugus*) survey data. Comparisons are made for 2016–2018 between vague priors (gray) and empirically informative priors (black)

**Figure 4:**
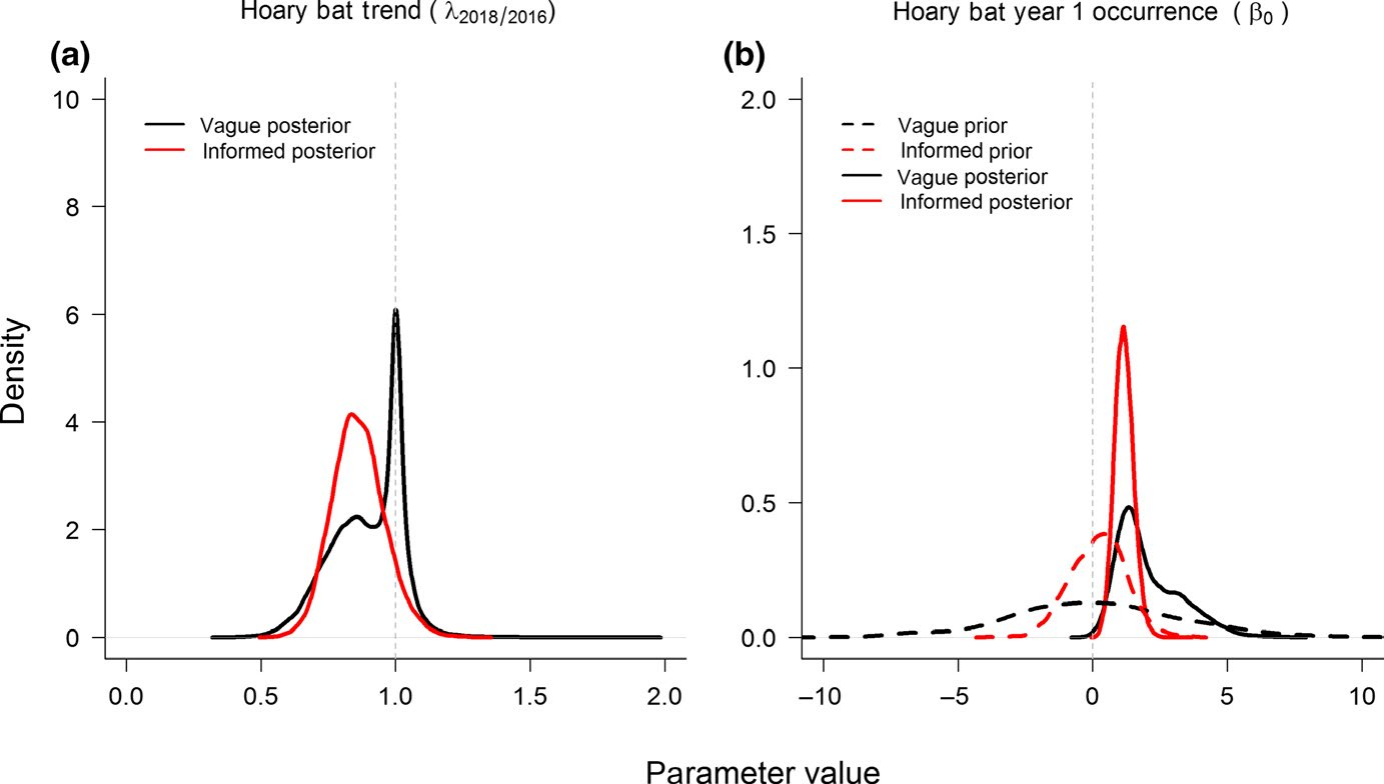
Comparison of empirically informed (red) and vaguely informed (black) priors and posteriors for hoary bat (left, a) trend and (right, b) year 1 occurrence probability (intercept parameter, logit scale; see Section 2 for auto‐logistic parameterization and use of Normal priors)

Our results provide evidence of decline in net summertime regional hoary bat occurrence probability during 2016–2018 relative to 2010 (Figure 3a) but no evidence of decline for the little brown bat (Figure 3b). These conclusions were supported by both the empirically informed and vague priors models (Figures 3 and 4). Choice of prior did not influence overall conclusions for trend although empirically informed priors provided more precise estimates (posterior probabilities with narrower 95% credible intervals; Figures 3 and 4) and therefore strengthened evidence of hoary bat decline. Estimates of trend ($\hat{\lambda }$) during 2016–2018 for hoary bat was 0.86 ± 0.10 (0.89 ± 0.12 when vague priors were used; Figure 4a), an average annual rate of decline since 2010, manifesting a ≈2%/year decline in net occurrence probability (i.e., from $\hat{\psi }$_2010_ = 0.87 to $\hat{\psi }$_2018_ = 0.65), and $\hat{\lambda }$ = 1.1 ± 0.10 (1.01 ± 0.10 when vague priors were used) for little brown bat. Detection probabilities were stable among years within each period but increased from ~25% for both species in Period 1 (see Rodhouse et al., 2015) to ~40% for hoary bat and ~50% for little brown bat in Period 2.

**Figure 5:**
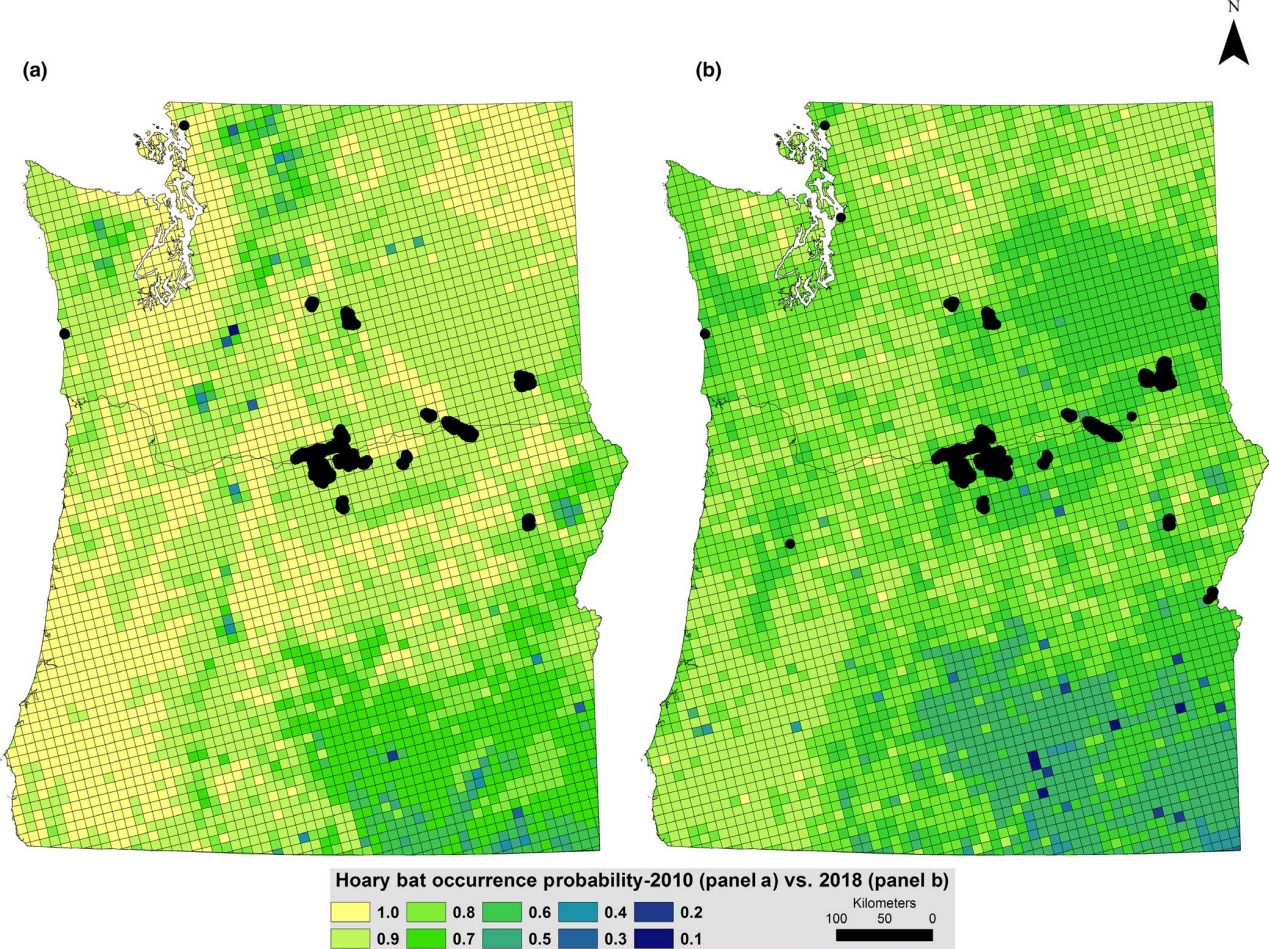
Comparative maps of 2010 (a, modified from Rodhouse et al., 2015) and 2018 (b) hoary bat predicted occurrence probabilities (${\hat{\psi }}_{i}$). Wind energy turbines (Hoen et al., 2018) are shown with black symbols circa 2010 in (a) and circa 2018 in (b). cf. with continent‐wide wind energy facility distribution at https://eerscmap.usgs.gov/uswtdb/ and also the Hayes et al. (2015) overlay of continental hoary bat seasonal migration distribution maps and wind facility distribution circa 2015

Mapped hoary bat occurrence predictions illustrated the overall net decline in the region for this species between 2010 and 2018 (Figure 5). Predictive performance of the 2018 hoary bat occurrence probability model, as measured by AUC posterior summary, was 0.80 (95% credible interval 0.74–0.86), an improvement over the 2010 predictions (AUC = 0.75) achieved by Rodhouse et al. (2015). For reference, we overlaid published wind turbine locations (Hoen et al., 2018) on our hoary bat occurrence probability maps which showed that development has not substantially increased since 2010 and that development is concentrated in the center of the study region along the breaks of the Columbia River along the Oregon/Washington border (Figure 5). We did not update predictive maps for little brown bat given the evidence of no change since 2010 in occurrence probability (flat trend; Figure 3b and λ ~ 1).

Inferences on the effect sizes of the environmental covariates forest cover, elevation, precipitation, and topographic roughness did not vary for either species in direction and magnitude between Period 1 and Period 2 nor between vague and empirically informed prior models (Appendix S1). However, precision of estimated effect sizes increased when informative priors were used, strengthening the influence of forest cover on hoary bat occurrence. Strength of evidence for the positive influence of precipitation on little brown bat occurrence also increased in Period 2, illustrated by the right shift along the *x* axis in Appendix S1 (Figure S2d).

## 4 DISCUSSION

We found evidence of decline for the summertime hoary bat population in the Pacific Northwest over the period 2003–2018, most notably since ~2007, but no evidence of decline during the same time period for the little brown bat. White‐nose syndrome was first reported in the region in 2016 but has not yet resulted in widespread regional impact to the little brown bat as has occurred in eastern North America (Frick et al., 2015). At the time of data collection (2016–2018), reports of the disease within our study region had not yet spread outside of the Puget Sound region of NW Washington and had not yet been reported in surrounding states (Idaho, Montana, Nevada, California). Wind energy development, however, is much more extensive in western North America (although not conspicuously so within our study region relative to other regions of North America; cf. Figure 5 and Hayes, Cryan, & Wunder, 2015) and is likely to have caused many hoary bat fatalities over a longer period of time (e.g., since ~2000; Arnett et al., 2016; O'Shea et al., 2016). We emphasize that model uncertainty (e.g., wide credible intervals in early years of study), bat longevity, a 5‐year gap in monitoring between Period 1 and Period 2, and only 3 years of additional data in Period 2 make these findings best considered as provisional evidence of decline that can guide conservation decisions, including the motivation to continue to allocate resources for further research and monitoring. However, given the laxity (curvature) in the occupancy–abundance relationship, evaluating population decline with occupancy models is inherently conservative, and our finding of hoary bat decline is alarming. Compelling empirical evidence of regional and range‐wide bat decline is difficult to obtain and rarely reported, and our study is unique in geographic and temporal extent, with evident implications for potential hoary bat extirpation risk proposed by Frick et al. (2017) if our observed hoary bat trend continues. Likewise, if WNS continues to spread throughout the region and exhibit the same levels of morbidity as has been reported from eastern North America then our monitoring and modeling framework, with many years of pre‐WNS prior information now available, provides the foundation for evaluating post‐WNS host population impacts as a replicated before–after impact study.

The evidence for hoary bat population decline and for species–environment relationships (i.e., hoary bats and forest cover and little brown bats and precipitation) provided by our study was strengthened when empirically informed priors were used. This is consistent with previous applications of informative priors to ecological research (e.g., Morris et al., 2015), and our study contributes a new demonstration of the utility of using informative priors to gain efficiencies in long‐term studies and monitoring. Historically, concerns were raised about the subjectivity and potential biases of using informative priors in Bayesian analyses that exerted too much influence on posterior distributions (e.g., Dennis, 1996), but with contemporary computing power, it has become straightforward to examine the influences of prior specification strategies (e.g., Dorazio & Johnson, 2003; Morris et al., 2015; Northrup & Gerber, 2018). Informative priors increase effective sample size (e.g., Hobbs & Hooten, 2015; McCarthy et al., 2005), and in our study, this benefit was realized by spanning the gap in data collection between 2010 and 2016. Data gaps are a common challenge for long‐term studies, and the improved ability to span gaps will be appealing to monitoring practitioners.

The overlay of wind turbine locations on our predictive hoary bat occurrence maps revealed that turbine density has not increased greatly over the course of study and, in general, is not very extensive relative to other regions of the country (cf. https://eerscmap.usgs.gov/uswtdb/viewer/). Hoary bat migration patterns are still not well described, and it remains unclear where the hoary bats that occur in our study region during summer monitoring are being killed (Cryan, 2003; Cryan & Brown, 2007; Hayes et al., 2015). Cryan (2003) and Hayes et al. (2015) developed maps of seasonal hoary bat occurrence patterns that suggest bats that occur in our region during summer could spend winters in and migrate through regions where turbine densities are much higher, offering a possible explanation for decline in the Northwestern United States. Although available evidence supports the working hypothesis that regional hoary bat decline is likely caused by elevated adult mortality from turbine collisions and barotrauma during fall migration, our results reflect net cumulative impacts, and a limitation of our study is the imprecision with which stressor impacts can be ascribed. In part, one solution to this limitation is to strive for broader regional and range‐wide replication of coordinated monitoring as advocated via NABat by Loeb et al. (2015) and using the modeling framework demonstrated here. A second solution will be to close the information gap about bat migration and other bat natural history using novel methods such as transmitter suturing developed by Castle, Weller, Cryan, Hein, and Schirmacher (2015) that has revealed long‐distance movements of hoary bats (Weller et al., 2016). A third solution will be to integrate geographically extensive coordinated acoustic surveys into a conservation information system that draws on multiple lines of evidence.

**Figure 6:**
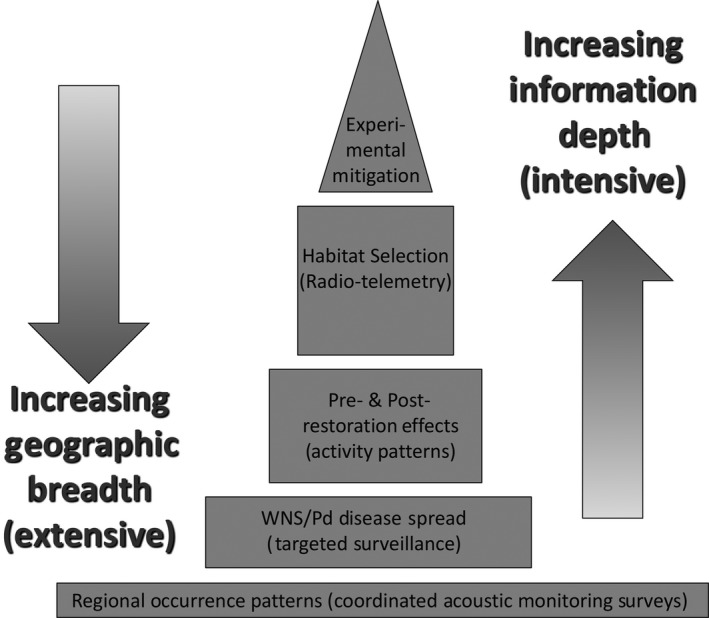
Conceptual diagram of an information pyramid that describes the inherent trade‐off between geographic extent and informational intensity of monitoring and supporting research that can be integrated into a rich model‐based information system for guiding evidence‐based bat conservation. Our geographically extensive monitoring from coordinated acoustic surveys and modeling of those data provides a robust “base” of the pyramid that can help identify when and where targeted and more informationally deep studies can be effective. Intensive local‐scale studies have been integrated into our grid‐based monitoring framework to simultaneously pursue local and regional objectives

Toward this third solution, we envision that our monitoring and modeling approach can provide the base of a strategic conservation information system “pyramid” (Figure 6), as has been done similarly through the integration of focal apex sites and broad‐scale occupancy modeling by the Amphibian and Reptile Monitoring Initiative (see https://armi.usgs.gov/program_design.php). Figure 6 illustrates the inherent trade‐offs in surveying across geographic extents with large sample sizes and depth of information content from more focused intensive study that can be ameliorated through strategic integration. For example, with respect to apparent hoary bat decline, our study, as a fundamental baseline, could be a catalyst for increased mitigation of wind turbine collisions via curtailment at low wind speed (Arnett, Huso, Schirmacher, & Hayes, 2011) and other actions (e.g., acoustic deterrence, Arnett, Hein, Schirmacher, Huso, & Szewczak, 2013). If done in a strategic manner (e.g., using experimental design), this can become a way to inform collective learning and adaptive management (Hayes et al., 2019). As another example, studies of the effects of forest thinning for forest fire fuels reduction on bats in the region's national parks (A. Chung‐MacCoubrey and S. Mohren, National Park Service, personal communication) have been nested within NABat grid cells, creating an opportunity for data collected during more‐informative but geographically less‐extensive focal studies to contribute simultaneously to our periodic region‐wide trend assessments. It is in this way that the coarse‐grained grid‐based NABat monitoring can become relevant at local‐scales, building bottom‐up engagement for a regional conservation program that requires top‐down coordination.

For the present study, region‐wide net hoary bat decline was hypothesized to be the result of fatalities at wind energy facilities outside the study region and during autumn (see Figure 4 in Hayes et al., 2015) unobserved by our study. We did not consider whether hoary bat occurrence trend over time might also co‐vary over space along, for example, forest cover or elevation gradients, but our framework could support pursuit of these questions, particularly if the energy facility footprint expands in the region along these environmental gradients (e.g., if predominantly in open agricultural and steppe landscapes) and compelling hypotheses about spatial variation in hoary bat decline are articulated. However, we find it more tangible at present that if WNS impacts on the little brown bat population become more widespread (i.e., from carcass recoveries throughout the region), a plausible hypothesis of an interaction between precipitation and little brown bat decline could be proposed because the disease has been reported to occur along precipitation and humidity gradients in eastern North America (Langwig et al., 2012) and our region has strong moisture gradients that may strongly influence disease spread and morbidity. This hypothesis could be evaluated with our empirical monitoring‐data‐model framework via inclusion of an interaction between the precipitation covariate (and other relevant covariates) and the dynamics of colonization and survival as b_t_*z(*i*,*t*−1) + *β*_3_Precipitation*_i_* + *β*_5_Precipitation_i_*z(*i*,*t*−1) (Royle & Dorazio, 2008).

In conclusion, empirically informed Bayesian modeling, fueled by large monitoring datasets that accumulate over time and that are underpinned by a robust survey design (e.g., our NABat spatially balanced master sample) provides a powerful and flexible foundation for building an adaptive, evidence‐based conservation information system. The long‐standing logistical challenges associated with studying bats that preclude directly estimating bat population sizes and demographic rates require the kinds of solutions that we demonstrate and discuss. Multiple lines of evidence, even if indirect, will be required to triangulate toward answers about the status and trends of bat populations.

## CONFLICT OF INTEREST

None declared.

## AUTHOR CONTRIBUTIONS

TJR, RMR, PCO, JB, and KMI designed and implemented the study. RMR coordinated region‐wide data acquisition. TJR conducted the modeling and KMB and KMI reviewed statistical procedures. TJR drafted the manuscript. All authors contributed to and edited the manuscript.

## Data Availability

The dataset and corresponding BUGS modeling code are archived on the National Park Service Integrated Resource Management Applications (IRMA) portal at: https://irma.nps.gov/DataStore/Reference/Profile/2264920.
