# Supplementary material for: Evidence of region‐wide bat population decline from long‐term monitoring and Bayesian occupancy models with empirically informed priors
Source: Ecol Evol. 2019 Sep 11;9(19):11078–88. doi: 10.1002/ece3.5612 (PMC6802066; doi:10.1002/ece3.5612)
Supplement: Supplementary file 1 [file ECE3-9-11078-s001.docx]

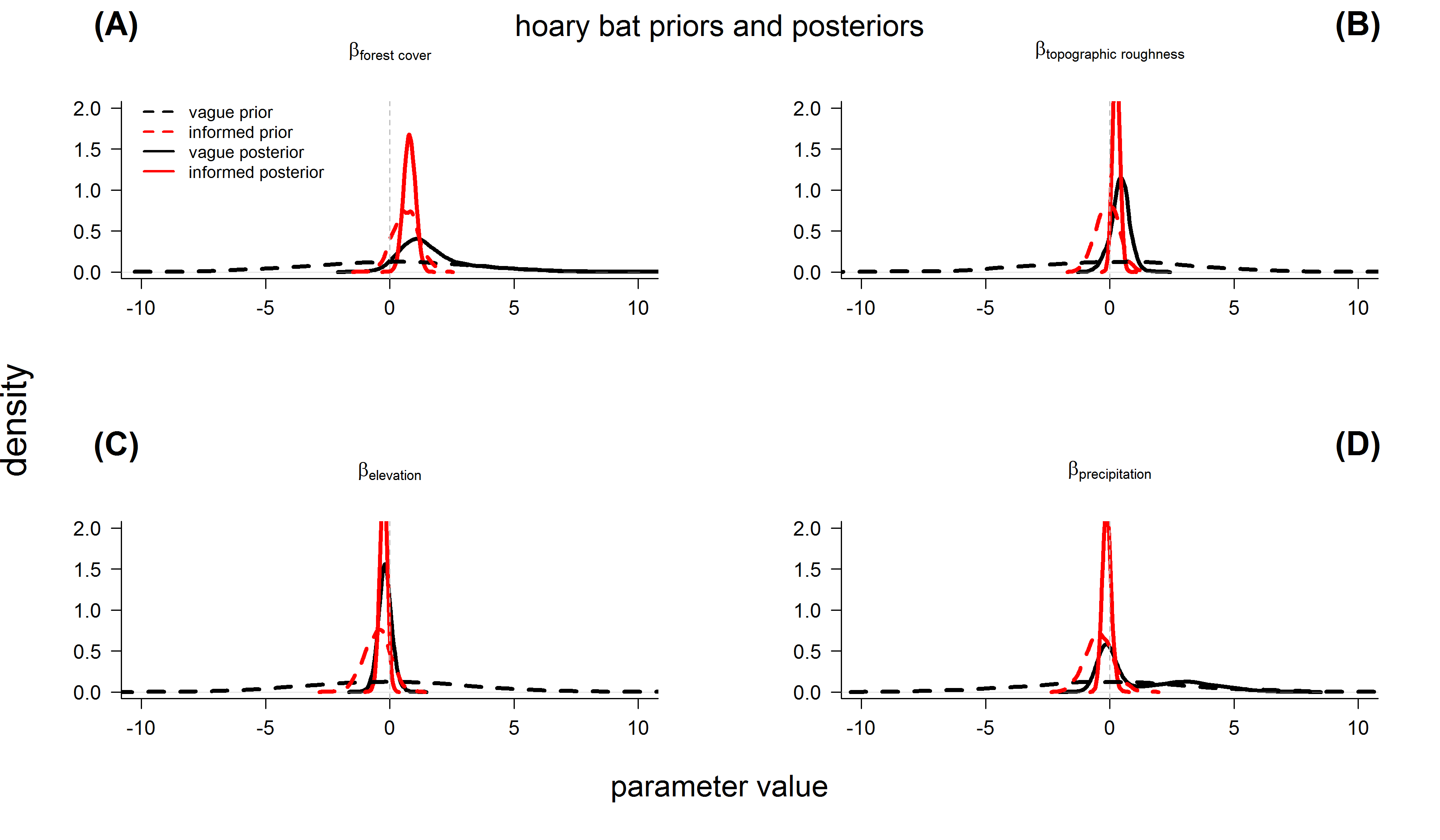


Figure S1. Priors and posteriors for environmental covariate parameters used to model hoary bat region-wide occurrence patterns. Panel A is forest cover (β_forest_); panel B is topographic roughness (β_topographic roughness_); panel C is elevation (β_elevation_); panel D is precipitation (β_precipitation_). In all 4 panels, precision increased with use of informative priors but inferences did not differ relative to use of vague priors. Increased precision strengthened evidence for the positive influence of forest cover on hoary bat occurrence.


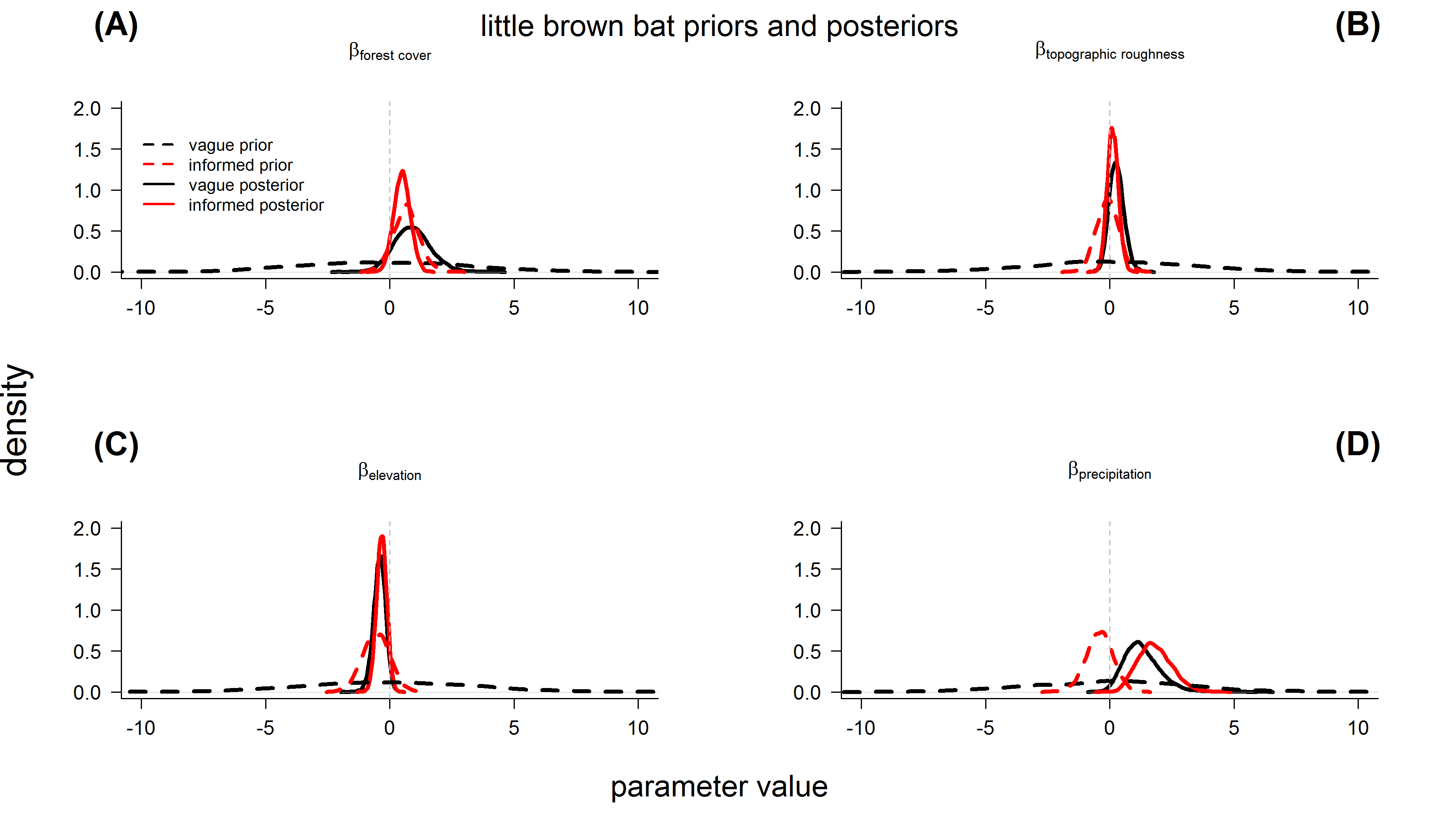


Figure S2. Priors and posteriors for environmental covariate parameters used to model little brown bat region-wide occurrence patterns. Panel A is forest cover (β_forest_); panel B is topographic roughness (β_topographic roughness_); panel C is elevation (β_elevation_); panel D is precipitation (β_precipitation_). Inferences did not differ relative to use of vague priors but precision increased for forest cover (panel A) and topographic roughness (panel B). Strength of evidence increased (right shift along the x-axis) in period 2 (2016-2018) for the positive influence of precipitation (panel D) on little brown bat occurrence relative to period 1 (2003-2010).
